# Electronic Health Records as an Educational Tool: Viewpoint

**DOI:** 10.2196/10306

**Published:** 2018-11-12

**Authors:** Yacob Habboush, Robert Hoyt, Sary Beidas

**Affiliations:** 1 Department of Internal Medicine Orange Park Medical Center HCA South Atlantic Division Orange Park, FL United States; 2 College of Allied Health Professions University of Nebraska Medical Center Omaha, NE United States

**Keywords:** electronic health records, education, teaching, learning

## Abstract

**Background:**

Electronic health records (EHRs) have been adopted by most hospitals and medical offices in the United States. Because of the rapidity of implementation, health care providers have not been able to leverage the full potential of the EHR for enhancing clinical care, learning, and teaching. Physicians are spending an average of 49% of their working hours on EHR documentation, chart review, and other indirect tasks related to patient care, which translates into less face time with patients.

**Objective:**

The purpose of this article is to provide a preliminary framework to guide the use of EHRs in teaching and evaluation of residents.

**Methods:**

First we discuss EHR educational capabilities that have not been reviewed in sufficient detail in the literature and expand our discussion for each educational activity with examples. We emphasize quality improvement of clinical notes as a basic foundational skill using a spreadsheet-based application as an assessment tool. Next, we integrate the six Accreditation Council for Graduate Medical Education (ACGME) Core Competencies and Milestones (CCMs) framework with the Reporter-Interpreter-Manager-Educator (RIME) model to expand our assessments of other areas of resident performance related to EHR use. Finally, we discuss how clinical utility, clinical outcome, and clinical reasoning skills can be assessed in the EHR.

**Results:**

We describe a pilot conceptual framework—CCM framework—to guide and demonstrate the use of the EHR for education in a clinical setting.

**Conclusions:**

As EHRs and other supporting technologies evolve, medical educators should continue to look for new opportunities within the EHR for education. Our framework is flexible to allow adaptation and use in most training programs. Future research should assess the validity of such methods on trainees’ education.

## Introduction

By July 2016, 95% [1] of hospitals and 60% [2] of office-based physicians had adopted electronic health records (EHRs). Because of the rapid adoption of EHRs, physicians may not have fully leveraged the potential benefits of using the EHR as a teaching tool to enhance medical education, clinical care, and efficiency [3].

Typically, physicians spend an average of 49% of their working hours using the EHR to document, review charts, and perform other indirect tasks related to patient care [4]. This translates into less face time with patients [4]. Given the significant amount of time physicians spend on EHR-related tasks, educators have an opportunity to help learners leverage the capabilities inherent to the EHR and thus improve the quality of patient care.

To achieve our aim, we incorporated the six Core Competencies and Milestones (CCMs) of the Accreditation Council for Graduate Medical Education (ACGME) into a framework to inform our teaching using the EHR. Our efforts expand on the Tierney et al [5] report, which focused on the ACGME’s Core Competencies and EHR tasks. Under this framework, we emphasize high-quality clinical notes as a foundational means to assess trainees’ activities in the EHR and correlate these activities to their level of training. We recognize that our framework may have limitations and it will evolve over time as EHR functionality and use benefit from technological improvements (eg, improved usability, input from data related to genomics, population health, and mobile phones).

**Figure 1:**
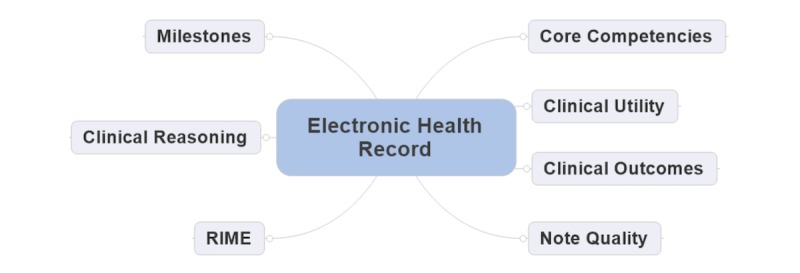
Electronic health record educational tools flowchart. RIME: Reporter-Interpreter-Manager-Educator.

In this article, we elaborate on using the EHR as a tool to enhance educational activities by mapping the tools in relation to the EHR as illustrated in Figure 1. We also discuss how different components, such as the ACGME’s CCMs, QNOTE, the Reporter-Interpreter-Manager-Educator (RIME) framework, clinical utility, clinical outcome, and clinical reasoning, are facilitated through the use of the EHR for the purpose of education.

We are not aware of other studies that have linked tasks and activities in the EHR to CCMs for the purpose of assessing education.

## Methods

In this article, we attempt to combine multiple educational concepts and tools to enhance the teaching and learning experience of medical students and residents. The following are the components used to focus on the assessment of trainees’ progression.

### Competencies and Milestones Integration

#### Overview

ACGME’s CCMs are a set of guidelines that allow a graduate medical education program to assess the progression of residents during clinical training. Monitoring trainees’ progression through EHR use can help in identifying gaps in knowledge, problem solving, and skills which can be targeted and remediated. For example, identifying a resident’s case-mix to ensure sufficient exposure during training in their specialty [6,7].

For integration of the CCMs, we used an internal medicine residency program to map the progression of an internal medicine resident through a longitudinal time frame. We have incorporated practicable links between the ACGME’s CCMs.

Next, we describe the ACGME’s Core Competencies with examples of activities a learner performs in the EHR that educators can use as an opportunity to enhance learning and evaluation.

#### Patient Care

Clinical notes have multiple stakeholders. To achieve a patient-centered stance in the EHR, patients should be able to read physician notes. Hence, we should insist that our trainees limit the use of abbreviations and avoid scientific jargon. This is especially relevant in the assessment and plan section of the note. Also, instructions should be succinct and clear and the use of complete sentences is encouraged to facilitate the understanding of the physician’s advice and recommendations. Patient-centered notes should also reflect a patient’s involvement in decision making through highlighting patient preferences in conjunction with evidence-based medicine (EBM) guidelines and protocols [8]. Avoiding altogether the use of the copy-and-paste function or relying on automated text to complete medical notes with standardized templates and auto-populated notes further degrades the quality of an EHR note [9,10]. A direct result from using these tools is “note bloat” [11]. There may be a place for automation in documentation when properly planned and executed; however, as it is fashionably used, automation adds insult to injury [5].

#### Medical Knowledge

Continuous learning and reinforcement learning within the EHR environment is a desirable behavior. This can be achieved by adding short EBM notes in the assessment and plan section of the clinical note. EBM tools embedded in the EHR or through clinical decision-support tools displayed at a well-planned strategic interface facilitate an EBM-centric documentation process (eg, using the 2017 American Heart Association guidelines to document the optimal blood pressure target of 130/80 mmHg in a patient whose blood pressure was 160/87 mmHg recorded during a follow-up clinic visit). Furthermore, the EHR can also be used during the morning preclinic brief where a multidisciplinary approach is implemented to promote preclinical resident preparation and encourage teamwork. Once the documentation of clinical notes is optimized—residents demonstrate high-quality notes, consistently—other EHR-related tools, such as alerts, drug interactions, and preventive guides, can further contribute to strengthening continuous learning habits [10,12].

#### Practice-Based Learning and Improvement

Data from the EHR can be extracted to a population health spreadsheet for use as an example of practice-based learning and improvement to expose trainees to a population-based approach. Thus, desired clinical measures can be tracked to support patient management. Population health is a core principle of measurement-based care where reimbursement is tied to value-based care. Practice-based learning and improvement activities achieved through the use of an EHR can support a continuous lifelong learning experience [13,14]. For instance, trainees could use the point-of-care EBM tools, such as UpToDate or DynaMed, to quickly read, access, and apply recommendations for clinical management of patients.

#### Interpersonal and Communication Skills

The “huddle” or “morning brief” requires that trainees prepare for the day and anticipate patients’ needs by reviewing scheduled patient charts. Other uses that facilitate interpersonal and communication skills include presenting cases during academic sessions, communicating with patients via a patient portal, and communicating with other staff members through an internal messaging system [15]. Setting up the exam rooms in a patient-centered stance supports interpersonal and communication skills. This can be achieved by installing a large monitor to access the EHR and display EHR content, which helps with patient education [16,17]. The use of computers during patient consultations is perceived positively by most patients if the physician takes into account the presence of a “third person” in the exam room and favorably adjusts their verbal and nonverbal communication behaviors [18,19].

From an educational perspective, the EHR can be used to identify the interactions taking place between team members. To illustrate, attending physicians can and do review the charts prior to interaction with trainees. This may eliminate the benefit of presenting findings, sparking rational discussions, and formulating an informative decision. Therefore, attending previsit charts may unintentionally compromise some aspects in the process of learning. One way to remedy this is to establish a daily morning brief where the whole team comes together to identify and review complex cases before clinic begins. Other interpersonal and communication skills benefits from the huddle include promoting team dynamics and interdisciplinary work [20].

#### Professionalism

The widespread adoption of EHRs has caused physicians to change how they interact with patients and staff [16]. Assessment of professionalism-related issues in the EHR may relate to incomplete notes, spelling and grammatical errors, note bloat, unsigned notes, organization, and structure. Appropriate balance between using the EHR and interacting with patients during clinical visits has been shown to increase interpersonal interaction with patients; hence, excessive time spent looking at displays rather than talking with patients is unfavorably perceived by patients [16,21]. Physicians’ professionalism also applies to communications with others for consultations and referrals, review of labs in timely manner, and communication of results to patients without the use of jargon, abbreviations, slang, or derogatory terms [2,17].

#### Systems-Based Practice

System-based practice refers to the process of providing cost-effective health care through integrating a team approach to patient care [22]. Examples of system-based practice in relation to the EHR include being able to identify safety errors or identifying quality-improvement gaps in the EHR. In addition, the EHR can provide outcome-based knowledge by analysis of specific population cohorts, such as frequent admissions, frail elderly, congestive heart failure, and diabetic mellitus [5] (ie, automated identification through the EHR of patients who need influenza and pneumococcal immunization to lessen comorbidities in eligible populations). Educators can assess notes for team members’ interactions, such as notes that demonstrate cohesion in assessment and plans.

Next, we discuss the remaining educational tools and concepts.

### Note Quality

QNOTE is a validated evaluation tool—Microsoft Excel spreadsheet format—used to assess medical documentation notes for quality, completeness, and efficiency [5,23]. Clinical notes can be assessed for clarity, conciseness, prevalence, organization, priority, and sufficiency of information documented [23]. The ability of QNOTE to generate a quantitative score for clinical notes assists with identifying the gaps in documentation so users learn how to properly document and provides users a sense of what they need to do to remediate their documentation skills. We have used QNOTE to assist residents in identifying gaps in clinical note documentation through a peer-to-peer EHR chart review. Although a structured note is desirable from a data-centric perspective, free text in clinical notes is necessary for context and storytelling [24]. For example, a *subjective, objective, assessment,* and *plan* note might be well structured with all components fully documented; however, the note may lack critical analysis and clinical reasoning where trainees fail to document their thinking process behind the assessment and plan section and patients’ preferences.

### Reporter-Interpreter-Manager-Educator Framework

RIME is an assessment framework used to evaluate trainees’ professional progression through four stages: Reporter, Interpreter, Manager, and Educator [25]. An EHR can provide educators with a feedback tool to monitor a trainee’s progression. For example, as trainees progress through the stages of RIME, they also progress in the stages of relationship to the team (ie, dependent, independent, and collaborative), level of performance (ie, reporter, interpreter, manager, and educator), and level of diligence. Here, diligence is defined as being comprehensive, paying careful attention, and consistently looking for information [26]. Within each stage of RIME, there are levels of expertise identified by an integration of knowledge, skills, diligence, team relationship, and performance level as illustrated by Cadieux and Goldszmidt [26]. Table 1 expands on RIME and provides examples for tracking trainees’ progression in the EHR [25,26].

### Clinical Utility

Trainees’ documentation needs to be assessed to ensure that the information collected from the patient is not only complete but also addresses identified patient problem (eg, a patient presents with a chief complaint of headache). In the system review, the resident finds out that the patient also has unintentional weight loss. Did the trainee make the connection between headache and weight loss? Was the differential diagnosis discussed in the note and was a plan of action clearly articulated and discussed with the patient [12,27,28]?

### Clinical Outcomes

The EHR could be used to assess clinical outcomes of patients managed by a particular resident after reviewing a longitudinal selected set of notes and labs to determine disease control versus progression. Furthermore, the EHR can be used to compare the disease progression of different patients diagnosed with similar diseases managed by different residents to assess the variation in care and if they meet the standards of care. Temporal data in the EHR can also uncover or forecast clinical outcomes [29]. For example, a diabetic patient’s complications trajectory could be tracked in the EHR by a resident to predict onset of end-stage renal disease. The EHR can also be used when presenting a clinical-case conference or a Morbidity and Mortality session— observing the Health Insurance Portability and Accountability Act and privacy rules by ensuring that individuals present are properly credentialed and patient consent for teaching purposes is documented—to assess outcomes by reviewing clinical notes directly from the EHR.

### Clinical Reasoning

During the first and second year of training, residents are developing their clinical reasoning documentation skills. Identifying the extent and depth for clinical reasoning skills is achieved by reading the assessment and plan section of the note. Residents can further enhance their clinical reasoning through learning how to synthesize and document a differential diagnosis and analyze the clinical information by identifying the key components in the patient’s history [30]. In addition, educators can look for instances when trainees are using heuristics (ie, intuitive thinking versus analytical thinking) in their clinical reasoning [31].

**Table 1:** Reporter-Interpreter-Manager-Educator model framework with examples.

Level of performance	Electronic health record feature	Examples from clinical notes
Reporter	Gather and document clinical facts Proficiency in history taking, physical examination, and basic medical knowledge Recognize normal from abnormal Answers “what” questions	“Patient with past medical history of diabetes presented to the emergency room complaining of chest pain. Pain started an hour ago while watching TV. It is crushing in character and located substernal. Pain does not radiate. There are no alleviating or exacerbating factors...”
Interpreter	Clinical reasoning Problem-solving skills Prioritize among problems identified and yield a differential diagnosis Follow up on diagnostic tests and analyze the data Minimal signs of collaborative team work Diligence Answer “why” questions	“...According to the CIDI^a^ 3 screening scale for bipolar disorder, the patient is at a very low risk with only one positive endorsement. Therefore, patient is unlikely to have bipolar disorder and more likely has depression. SSRI^b^ is initiated to manage his depression. Risk and benefits are explained to the patient; patient understands. Follow up in two weeks.”
Manager	Anticipate outcomes Independent decision-making process Provide alternative options Personalize assessment and plan Balance between team-dependent and team-independent relationship Patient centered Diligence Answers “how” questions	“...A1c is 11.4, insulin was recommended for the patient; however, after a collaborative decision, patient refused to start on insulin and preferred to initiate metformin and lifestyle adjustments.”
Educator	Self-directed learning Document teaching point Seek answers based on evidence-based medicine Share experiences and educational points Diligence	“According to the new ACC/AHA^c^ guidelines, patient’s blood pressure is at an optimal level of 124/78 mmHg. New guidelines have changed the target BP^d^ to lower than 130/80 rather than 140/90.”

^a^CIDI: Composite International Diagnostic Interview.

^b^SSRI: Selective serotonin reuptake inhibitor.

^c^ACC/AHA: American College of Cardiology/American Heart Association.

^d^BP: blood pressure.

**Figure 2:**
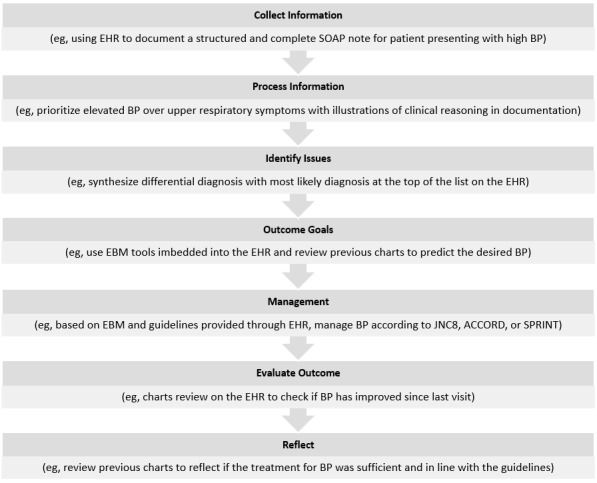
Clinical reasoning process with examples. EHR: electronic health record; SOAP: subjective, objective, assessment, and plan; BP: blood pressure; EBM: evidence-based medicine; JNC8: Eighth Joint National Committee; ACCORD: Action to Control Cardiovascular Risk in Diabetes; SPRINT: Systolic Blood Pressure Intervention Trial.

Clinicians can propose a diagnostic plan and desired outcome state of the patient; this will identify the necessary investigational tests and management plan to transit the patient from the present state to the outcome state after implementing the management plan through the decision-making process. Providing feedback to residents and giving them space to reflect on and narrow their differential diagnosis will help in improving the decision-making process and clinical thinking [30,32]. Figure 2 provides more examples for each of the stages in the clinical reasoning process.

## Results

**Figure 3:**
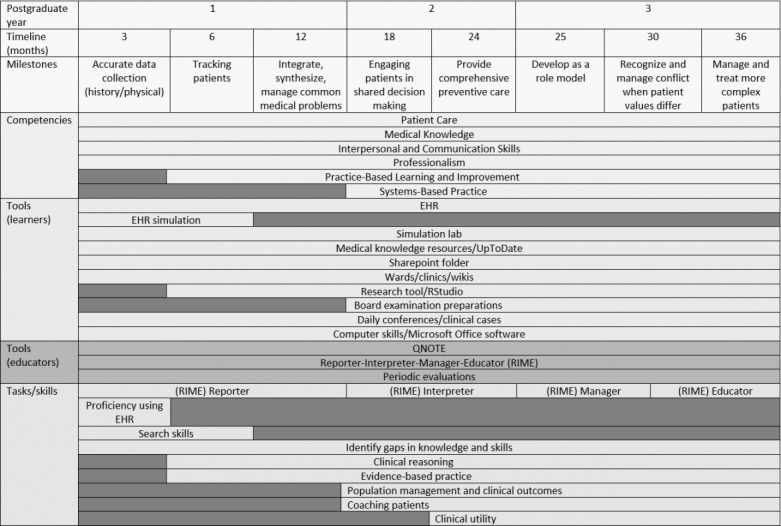
Pilot conceptual framework. EHR: electronic health record; RIME: Reporter-Interpreter-Manager-Educator.

We created a pilot conceptual framework (see Figure 3) to serve as a visual guide for accessing resident progression during training from an EHR perspective. Preliminary results from using our framework are supportive in continuing our course; however, our data are limited and incomplete.

The following is a demonstration of how to use the conceptual framework. We expect an intern to achieve proficiency by the end of the initial 3 months in the following tasks and skills: review documentations in the EHR, present a case to the attending physician, and use point-of-care knowledge applications like UpToDate or DynaMed Plus. For assessment purposes, we recommend using QNOTE for assessing note quality. QNOTE uses a spreadsheet form to assess 12 elements in the quality of clinical notes and quantitatively measures clinical documentation in the EHR [23]. Thus, connecting the intern’s proficiency in use of the EHR can be mapped to a Reporter performing level using the RIME model [25]. In addition, a Reporter is expected to be proficient in placing orders, retrieving labs and diagnostic images, documenting notes, and search skills. Reporters can sometimes reflect on their own performance and identify gaps in their clinical knowledge. Some may consider these activities as evidence of proficiency in using an EHR.

**Figure 4:**
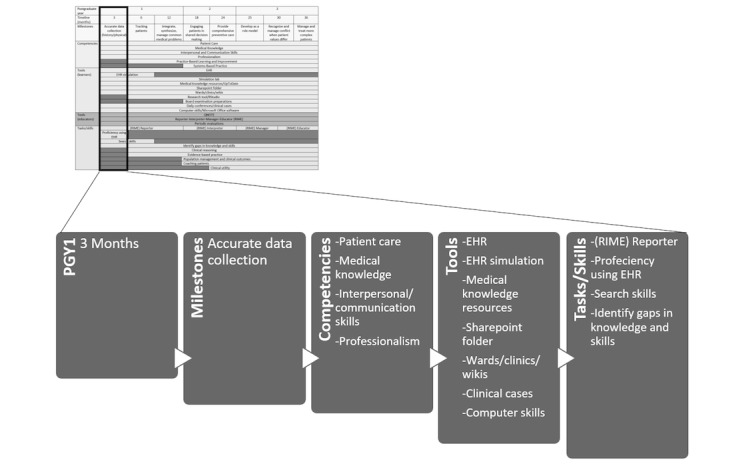
Example of how the conceptual framework in Figure 3 is utilized. PGY1: postgraduate year 1; EHR: electronic health record; RIME: Reporter-Interpreter-Manager-Educator.

In Figure 4, we illustrate by example how to use the CCMs conceptual framework by specifically focusing on the first 3 months of residency and linking the necessary milestones and competencies to the available technology to achieve the set tasks and skills.

## Discussion

During our search to uncover new ways to leverage the use of the EHR for teaching and learning, we found it advantageous to associate ACGME’s CCMs to EHR tasks and activities. Consequently, we developed the CCMs conceptual model (see Figure 4) as a guide to help in assessing residents as they progress through the residency program. The tools, tasks, and skills used in the CCMs framework are flexible and can be customized to address specific gaps in learning or assessment.

We emphasize the quality of clinical notes as a critical aspect of the CCMs framework. At least two retrospective studies examined physicians’ quality of notes and concluded that the EHR can improve the overall quality of notes [33,34]. Clinical notes can be assessed by an educator, or using peer-to-peer assessment, to provide direct feedback to trainees. Other studies have also shown benefits in utilizing EHR simulations, where trainees could learn how to accurately document clinical notes with constructive feedback to improve health care delivery while also identifying gaps in trainees’ knowledge and skills [35,36]. We believe EHR simulations are important tools in teaching sound EHR practices; however, they are beyond the scope of this article. Similar to educators in other fields, clinicians interested in teaching need to develop their teaching skills. Typically, faculty development programs have been the main venue for learning these skills. Other venues include university-degree granting programs in clinical education for those who are interested or have the time and resources to pursue a higher degree in medical education.

In the pre-EHR era, clinical hypo-competence was described as the lack of accurate information, failure to generate relevant differential diagnosis, and incomplete analysis and processing of information with a lack of a collaborative patient-centered approach. In this technology-driven era of health care we find that clinical hypo-competence remains as a persistent problem promoted by note bloat, use of macros, and automated sentence and phrase generation in EHRs [7]. Recognizing the importance of the EHR in today’s clinical environment, coupled with the rapid advances in technology, trainees, including medical students, should be exposed to clinical information systems (eg, EHRs, patient portals, devices, wearables, and other clinical technologies) as early as possible in their education to prepare them for the lifelong learning experience required for a successful career in medicine. We believe that clinical educators can assist trainees to develop a solid foundation in clinical documentation using the EHR and enhance proper use of applications for documentation, such as natural language processing tools and macro generators, to lessen trainees’ cognitive load and improve time-on-task processes when using clinical information systems.

The use of the EHR as an educational tool can facilitate clinic workflow, monitor trainees’ learning experience, improve clinical reasoning, and identify the gaps in trainees’ knowledge by mapping trainees’ progression through the ACGME’s CCMs [5,37]. Determining what cases trainees have experienced during a rotation could also be monitored through the EHR. A recent study reported that only 3% of postgraduate-year-1 residents are exposed to the 10 most common diagnoses, while 31% had experienced fewer than five of the diagnoses [34]. An EHR could be used to enhance research and quality-improvement projects through identifying certain populations to study. An EHR could also help us understand diseases and management plans and support better population health [38].

The limitations of this study include the experience of one institution. There is a paucity of published articles pertaining to using the EHR as a teaching tool. Major strengths of the study include the use of the standardized ACGME’s CCMs and validated tools such as QNOTE and the RIME framework. Also, the conceptual framework could be generalized and customized (see Figure 4) to most graduate medical education programs as it could be adopted by any program to assess trainees’ progression from a technology perspective.

In summary, physicians spend a significant portion of their working hours in the EHR. Clinical educators should continue to look for opportunities to uncover new approaches for education and use of the EHR in educational activities that benefit patient care, efficiency, and proper safe use. Our center is currently in the process of validating this conceptual framework through a prospective, institutional review board-approved study.

## Abbreviations

ACC/AHA: American College of Cardiology/American Heart Association
ACCORD: Action to Control Cardiovascular Risk in Diabetes
ACGME: Accreditation Council for Graduate Medical Education
BP: blood pressure
CCMs: Core Competencies and Milestones
CIDI: Composite International Diagnostic Interview
EBM: evidence-based medicine
EHR: electronic health record
JNC8: Eighth Joint National Committee
PGY1: postgraduate year 1
RIME: Reporter-Interpreter-Manager-Educator
SOAP: subjective, objective, assessment, and plan
SPRINT: Systolic Blood Pressure Intervention Trial
SSRI: Selective serotonin reuptake inhibitor
